# Cryopreservation and Validation of Microfragmented Adipose Tissue for Autologous Use in Knee Osteoarthritis Treatment

**DOI:** 10.3390/ijms26146969

**Published:** 2025-07-20

**Authors:** Marija Zekušić, Petar Brlek, Lucija Zenić, Vilim Molnar, Maja Ledinski, Marina Bujić Mihica, Adela Štimac, Beata Halassy, Snježana Ramić, Dominik Puljić, Tiha Vučemilo, Carlo Tremolada, Srećko Sabalić, David C. Karli, Dimitrios Tsoukas, Dragan Primorac

**Affiliations:** 1 Department of Transfusion and Regenerative Medicine, Sestre Milosrdnice University Hospital Center, 10000 Zagreb, Croatia; marija.zekusic@kbcsm.hr (M.Z.); maja.ledinski@kbcsm.hr (M.L.); marina.bujic@kbcsm.hr (M.B.M.);; 2 Faculty of Biotechnology and Drug Development, University of Rijeka, 51000 Rijeka, Croatia; 3 Department of Molecular Biology, Faculty of Science, University of Zagreb, 10000 Zagreb, Croatia; 4 St. Catherine Specialty Hospital, 10000 Zagreb, Croatia; 5 School of Medicine, Josip Juraj Strossmayer University of Osijek, 31000 Osijek, Croatia; 6 Greyledge Europe Ltd., 10000 Zagreb, Croatia; lucijazenic@greyledgebiotech.com; 7 Centre for Research and Knowledge Transfer in Biotechnology, University of Zagreb, 10000 Zagreb, Croatiabhalassy@unizg.hr (B.H.); 8 Department of Pathology and Cytology ‘Ljudevit Jurak’, Sestre Milosrdnice University Hospital Center, 10000 Zagreb, Croatia; 9 Image Regenerative Clinic, 20122 Milan, Italy; carlo.tremolada@gmail.com; 10 Department of Traumatology, Sestre Milosrdnice University Hospital Center, 10000 Zagreb, Croatia; 11 Medical School, University of Split, 21000 Split, Croatia; 12 Greyledge Technologies, LLC, Miami, FL 33133, USA; davidkarli@greyledgebiotech.com; 13 Orthopaedic Clinic for Advanced Arthroscopic Sports and Regenerative Surgery Mitera Hospital, 15123 Athens, Greece; info@tsoukas-ortho.gr; 14 Forensic Science Program, Department of Biochemistry & Molecular Biology, The Pennsylvania State University, State College, PA 16802, USA; 15 The Henry C. Lee College of Criminal Justice and Forensic Sciences, University of New Haven, West Haven, CT 06516, USA; 16 Sana Kliniken Oberfranken, 96450 Coburg, Germany; 17 Medical School, University of Rijeka, 51000 Rijeka, Croatia; 18 Faculty of Dental Medicine and Health, Josip Juraj Strossmayer University of Osijek, 31000 Osijek, Croatia; 19 Medical School, University of Mostar, 88000 Mostar, Bosnia and Herzegovina; 20 National Forensic Sciences University, Gandhinagar 382007, India

**Keywords:** MFAT, adipose tissue, endothelial progenitor cell, pericyte, osteoarthritis

## Abstract

Micro-fragmented adipose tissue (MFAT) is a promising autologous therapy for knee osteoarthritis. To avoid repeated liposuction procedures for its clinical application, MFAT obtained from patients with knee osteoarthritis was stored at −80 °C in a tissue bank. This study describes the preparation, cryopreservation, thawing, and washing, as well as comprehensive analysis of cell populations in fresh and MFAT thawed after two years. Immunophenotyping of both fresh and thawed MFAT showed a significant presence of endothelial progenitors and pericytes in the stromal vascular fraction. Viability before (59.75%) and after freezing (55.73%) showed no significant difference. However, the average cell count per gram of MFAT was significantly reduced in thawed samples (3.00 × 10^5^) compared to fresh ones (5.64 × 10^5^), likely due to processing steps. Thawed MFAT samples showed increased CD73 expression on the CD31^high^CD34^high^ subset of EP and SA-ASC, as well as increased expression of CD105 on EP, the CD31^low^CD34^low^ subset of EP, pericytes, and SA-ASC. Microbiological testing confirmed 100% sterility, and double washing efficiently removed DMSO, confirming sample safety. Histological analysis revealed healthy, uniformly shaped adipocytes with intact membranes. This approach allows accurate estimation of cell yield for intra-articular injection, ensuring delivery of the target cell number into the knee. Quality control analysis confirms that cryopreserved MFAT retains high cellular and structural integrity, supporting its safety and suitability for clinical application.

## 1. Introduction

Osteoarthritis (OA) is the most common progressive inflammatory disease affecting musculoskeletal tissues, primarily the hip and knee joints in humans. Recent preclinical and clinical studies have demonstrated that adipose tissue (AT)-derived therapies, including microfragmented adipose tissue (MFAT), have the potential for cartilage regeneration, inflammation reduction, and improvement of joint function [1]. Due to its accessibility and use as an autologous tissue source, AT is applied in regenerative medicine with minimal ethical concerns [2]. In patients with OA, AT can be collected under local anesthesia and then applied in a single surgical procedure or stored in a tissue bank for periodic use as needed. Currently, the Coleman technique is the most commonly used method for the procurement and transplantation of AT [3]. AT is typically collected from the abdomen using lidocaine, with a preference for the superficial layer of subcutaneous fat due to its higher stromal component content, ensuring high multipotency and regenerative potential. MFAT is mechanically processed AT that is microfragmented (0.2–0.8 mm), with preserved stem/stromal cells retained in their niches [4,5]. The application of fresh MFAT in patients with various stages of OA has resulted in improved symptoms, better knee function, and reduced pain, thereby delaying the need for invasive surgical procedures [6].

For the clinical application of fresh AT, the process should involve minimal manipulation, be intended for homologous use, and the surgical procedure should be carried out on the same day [7]. In order to enable clinicians to apply AT periodically over an extended period, cryopreservation and storage in a tissue bank are highly useful, eliminating the need for repeated liposuction procedures.

The advantages of using AT include its abundance, ease of procurement, and consistency, as it can be obtained through lipoaspiration, which is less invasive procedure compared to aspiration of bone marrow, another common source of mesenchymal stem/stromal cells (MSCs) [2,8]. Although the primary function of AT is to serve as an energy reserve stored by adipocytes, it also contains a heterogeneous cell population known as the stromal vascular fraction (SVF). Some of these cells have exceptional regenerative properties, which have attracted the attention of the scientific and medical communities [9]. SVF includes macrophages, endothelial cells, pericytes, and AT-MSCs [4,10]. This is crucial because, while MSCs can be obtained from multiple tissues, the isolation process often destroys tissue and cellular niches. However, the nature of AT, its structure and composition, enables the preservation of cellular niches during processing. This preservation is critical for regenerative purposes and the desired effects provided by MSCs through their paracrine mechanism of action [4]. In MSCs, the secretion of extracellular vesicles, which are rich in various biomolecules such as nucleic acids, proteins, lipids, and sugars, is crucial for immune modulation, intercellular signaling, and tissue repair, holding great therapeutic potential [4,5].

The cryopreservation process has to minimally affect MFAT to ensure its quality and safety for clinical application [11]. The cytotoxicity of cryopreservation is associated with two key processes: an increase in extracellular osmolarity and the formation of ice crystals within the cells. Ice crystals can cause cryoinjury and lead to cell death. These cytotoxic effects can be avoided through gradual freezing, rapid thawing, and the use of cryoprotectants [12]. To safely cryopreserve AT for long-term storage at −80 °C, various cryoprotectants can be used, including dimethyl sulfoxide (DMSO), trehalose, glycerol, or their combination [2]. While DMSO is effective in preserving the viability of cryopreserved cells, many side effects related to its use have been reported [11], along with its toxic activities [13]. With the development of sensitive high-throughput techniques and new areas of research, it has become evident that DMSO is not as inert as previously thought and may induce alterations in miRNAs and the epigenetic landscape [14]. Therefore, its use should be avoided when possible [15]. Although trehalose and glycerol are less toxic compared to DMSO, MSCs derived from lipoaspirates cryopreserved in DMSO exhibit better post-thaw growth, reaching confluence faster and maintaining their characteristic morphology compared to MSCs derived from lipoaspirates cryopreserved in trehalose [16]. Additionally, cryopreserved AT stored for up to three years in vapor-phase liquid nitrogen has been shown to retain its differentiation potential and viable precursors, making it promising for autologous transplantation [11].

In this study, we aimed to assess the safety, purity, and quality of cryopreserved autologous MFAT for its periodic clinical application in patients with knee OA. To the best of our knowledge, the clinical application of cryopreserved AT is a novel approach in the treatment of OA, with no standardized protocols currently available. Therefore, a detailed description of our method may benefit professionals in other tissue banks and surgeons applying this therapy in routine practice. One of our goals was to analyze microbiological sterility throughout all stages of tissue processing, namely before freezing, after freezing (stored for about two years at −80 °C), and immediately before clinical application as a part of the safety assessment of the MFAT preparation. Finally, to our knowledge, a method for washing MFAT to remove DMSO, as part of ensuring its purity, has not yet been described. By outlining the processing method and conducting quality control of cryopreserved MFAT, we aim to provide a high-quality and safe MFAT preparation that ensures effective therapy in routine clinical practice.

## 2. Results

### 2.1. Immunophenotyping of MFAT Samples

**Figure 1:**
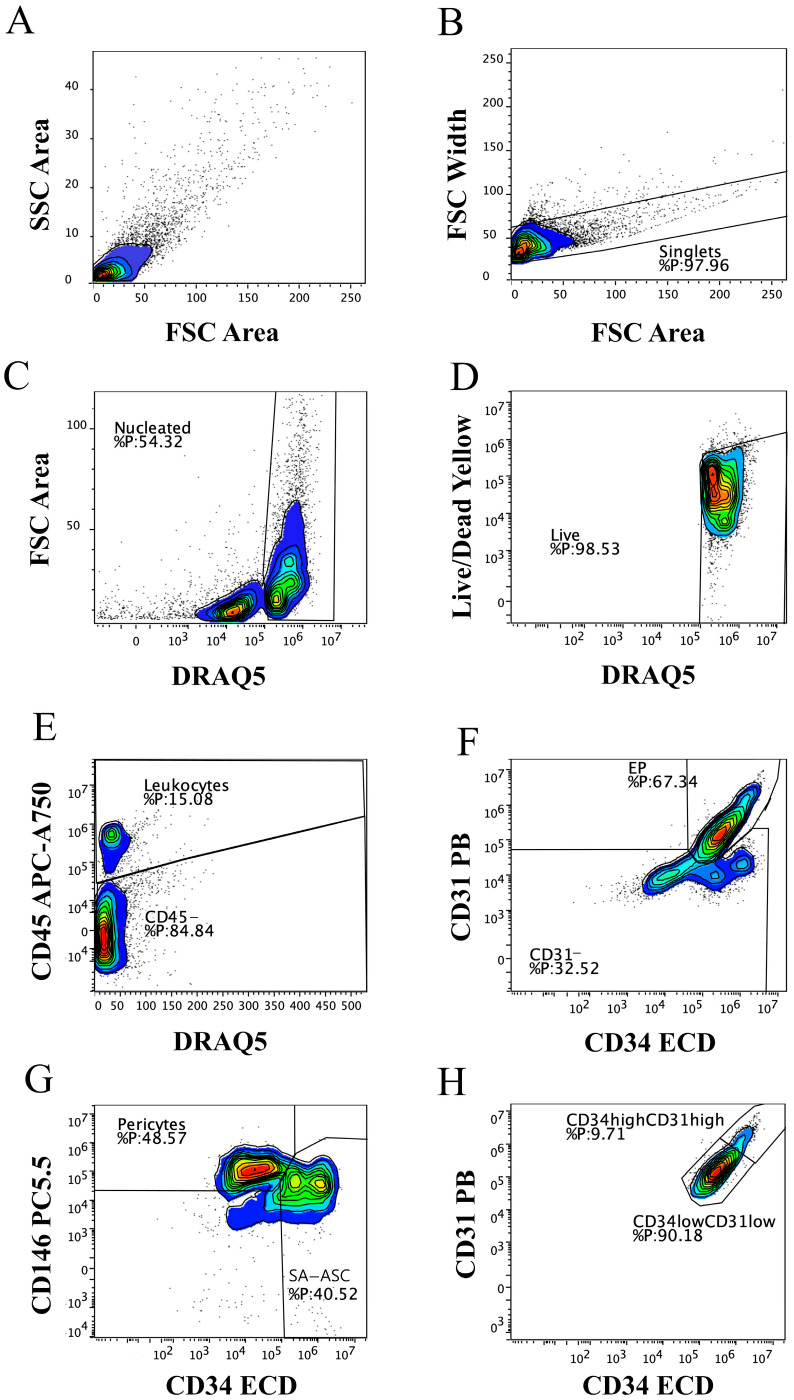
The gating protocol for the polychromatic flow cytometry analysis of the stromal vascular fraction from fresh microfragmented adipose tissue (MFAT). Forward Scatter (FSC) Area/Side Scatter (SSC) Area plot (**A**); singlet events based on forward scatter Area/Width (**B**), nucleated cell events selected by the DNA-binding DRAQ5 dye-positivity/FSC Area (**C**), and viable cells determined by the Live/Dead Yellow staining (**D**) were used to analyze the CD45^+^ and CD45^−^ cells (**E**). Nucleated live CD45^−^ cells were phenotyped according to the CD31 and CD34 lineage markers as CD31^+^ CD34^+^ endothelial progenitors (EP) and CD31^−^ non-endothelial population (**F**), which was, in combination with the CD146 marker, further phenotyped as pericytes and supra-adventitial adipose stromal cells (SA-ASC) (**G**). Based on the CD31 and CD34 marker intensity, EP can be discerned as CD31^high^CD34^high^ and CD31^low^CD34^low^ cell subpopulations (**H**).

We first performed a polychromatic flow cytometry immunophenotyping analysis in order to define the cellular composition of fresh MFAT (Figure 1). Based on our previous studies [17,18], we designed the gating protocol for selecting nucleated live cells (Figure 1A–D) and identifying MFAT immunophenotypes (Figure 1E–H). Using this gating strategy, we determined the main phenotypes of the CD45^+^ and CD45^−^ cell fractions in MFAT samples: CD45^+^CD31^−^CD34^−^CD146^−^ leukocytes, CD45^−^CD31^+^CD34^+^CD146^±^ endothelial progenitors (EP), with its CD45^−^CD31^low^CD34^low^CD146^±^ EP and CD45^−^CD31^high^CD34^high^CD146^±^ EP subsets, CD45^−^CD31^−^CD34^−^CD146^+^ pericytes, and CD45^−^CD31^−^CD34^+^CD146^−^ supra-adventitial adipose stromal cells (SA-ASC).

**Figure 2:**
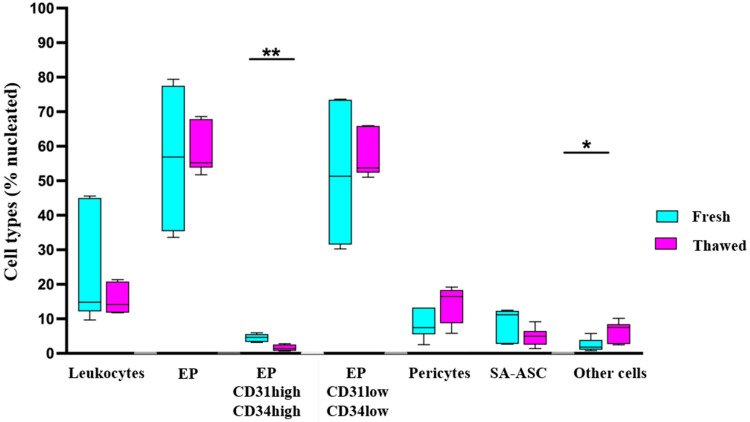
Flow cytometry immunophenotyping of cell populations in fresh and thawed MFAT samples—relative numbers. The ratio of each cell population is expressed as a percentage of live nucleated events in the samples from six patients before and after cryopreservation (EP = endothelial progenitors; SA-ASC = supra-adventitial adipose stromal cells). Statistical analysis was performed using a *t*-test based on the normality distribution; * *p* < 0.05, ** *p* < 0.01.

The same gating strategy was applied to the MFAT samples thawed after about two years in order to compare the effect of cryopreservation on the immunophenotype. The relative ratios of the cell populations, identified by immunophenotyping as shown in Figure 1, were calculated as a percentage of live nucleated events in fresh and thawed MFAT samples (Figure 2). The cells not identified by immunophenotyping due to the lack of additional identification markers were designated as “other cells”, and their relative and later absolute numbers accounted for the remaining amount to 100%.

**Figure 3:**
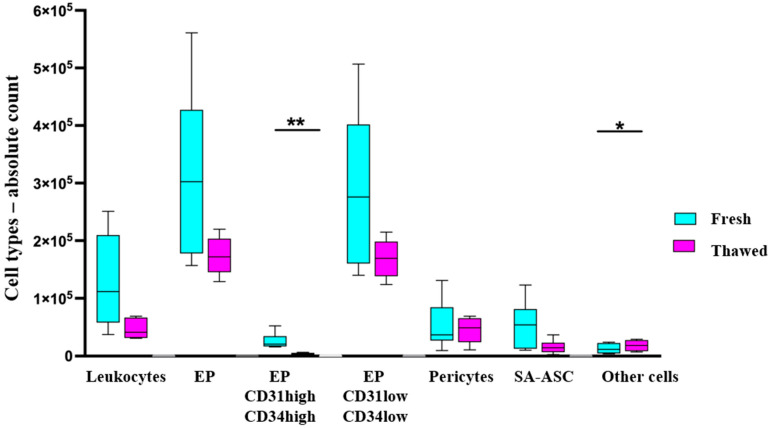
Flow cytometry immunophenotyping of cell populations in fresh and thawed MFAT samples—absolute numbers. The absolute count of each cell population is presented as a count per MFAT tissue gram of live nucleated events in the samples from six patients before and after cryopreservation (EP = endothelial progenitors; SA-ASC = supra-adventitial adipose stromal cells). Statistical analysis was performed using a *t*-test based on the normality distribution; * *p* < 0.05, ** *p* < 0.01.

The same six patient samples were analyzed for the total cell count, as well as counts of the specific cell populations identified by immunophenotyping as shown in Figure 1, presented as the number of cells per MFAT tissue gram (Figure 3).

### 2.2. Total Cell Viability and Count

**Figure 4:**
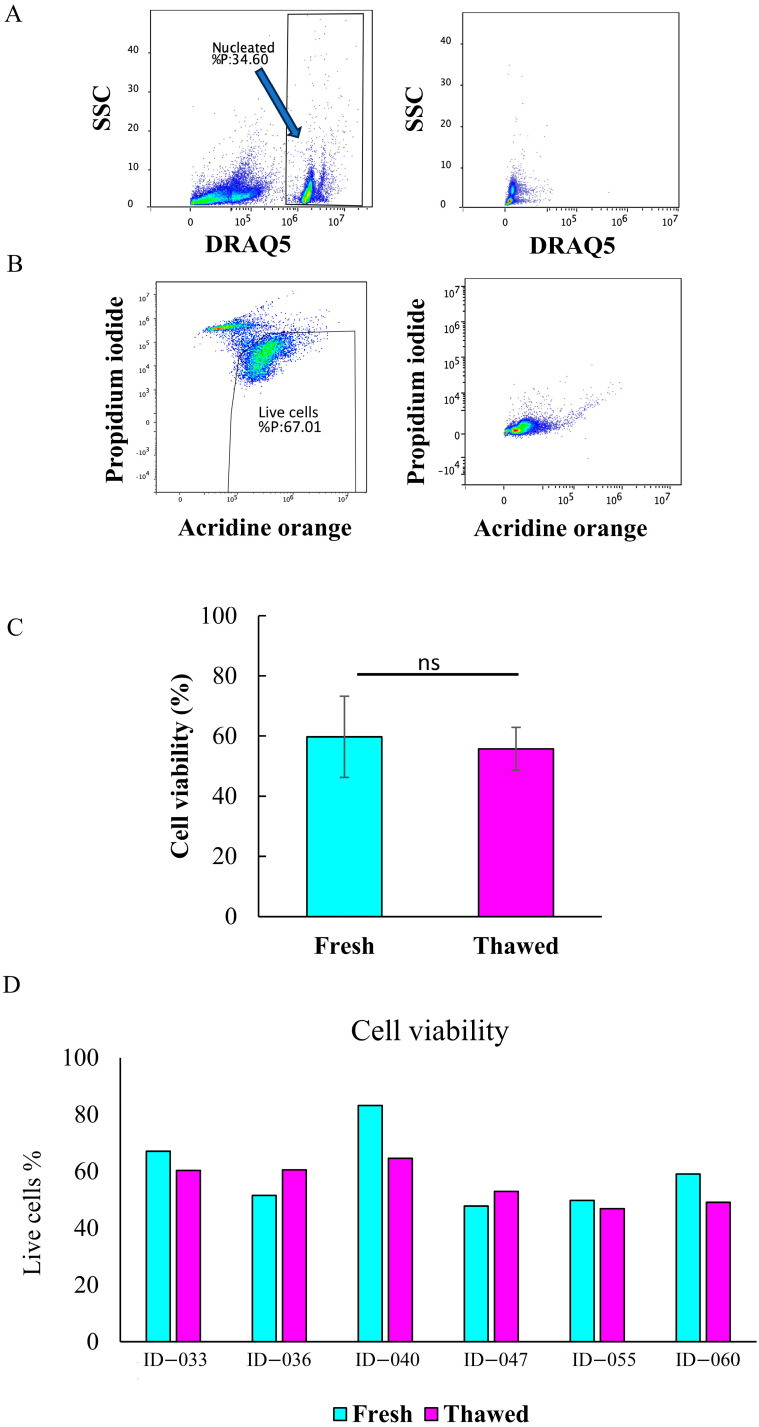
Cell viability of fresh and thawed MFAT samples. Gating strategy for viability determination (one representative experiment of a fresh sample is shown). Nucleated events were selected on the DRAQ5 vs. side scatter (SSC) density plot ((**A**), left panel); unstained control ((**A**), right panel). Only events from nucleated cells were analyzed further for viability, based on the DNA binding of acridine orange (AO) and propidium iodide (PI). Cells with the higher AO signal and lower PI signal were designated as live cells ((**B**), left panel); unstained control ((**B**), right panel). Mean difference in the percentage of live cells between fresh and thawed samples (*n* = 6) (**C**); paired *t*-test, ns = not significant. The percentage of live cells in individual fresh and thawed samples from six patients with ID numbers is shown (**D**).

**Figure 5:**
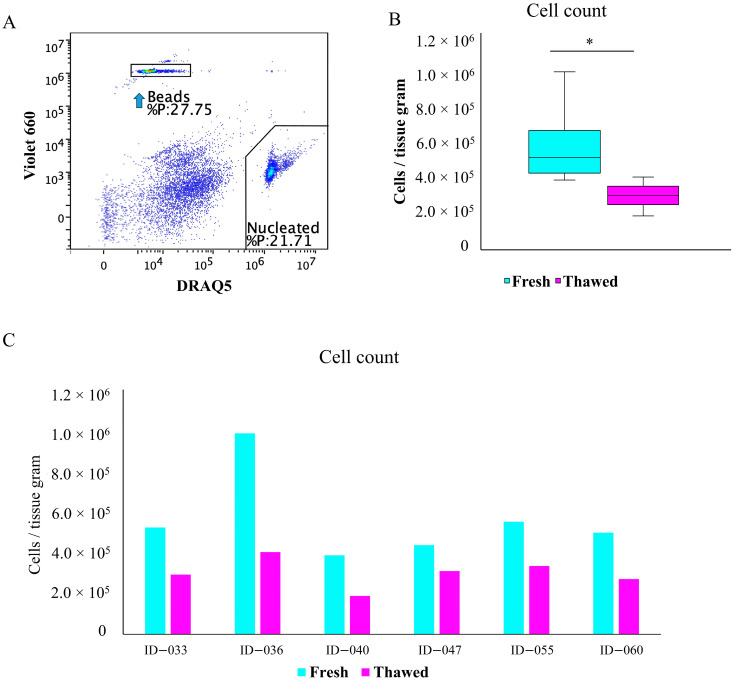
Cell count of fresh and thawed MFAT samples. Gating strategy for cell count determination. Nucleated events and count beads were selected on the DRAQ5 vs. Violet 660 density plot (**A**). Mean difference in the cell count of live cells between fresh and thawed samples (**B**) (n = 6); unpaired *t*-test, * *p* < 0.05. The cell count per gram of MFAT tissue in fresh and thawed samples from six patients with ID numbers is shown (**C**).

We next investigated the influence of cryopreservation on the total cell viability (Figure 4) and cell count (Figure 5) by flow cytometry. The gating strategy for identifying live nucleated cells was based on the DRAQ5 staining (Figure 4A), as well as the acridine orange (AO) and propidium iodide (PI) staining (Figure 4B). There was no statistically significant difference between the mean percentage of live cells in fresh (59.75%) and thawed (55.73%) samples (Figure 4C), indicating that the cell viability did not change in the course of cryopreservation. As shown in Figure 4D, the percentage of live cells in six fresh samples was between 47.80 and 83.20%, while that of the post-thaw counterparts ranged from 46.88 to 64.61%.

The gating procedure for determining the cell number based on the flow cytometric analysis using FlowCount fluorospheres and nucleated DRAQ5-positive events is shown in Figure 5A. A statistically significant difference was observed between the mean number of cells in fresh (5.63 × 10^5^) and thawed (3.00 × 10^5^) samples (Figure 5B), indicating a loss of approximately 50% of total cells. As shown in Figure 5C, the cell count in fresh samples ranged from 3.87 × 10^5^ to 9.85 × 10^5^, while in thawed samples it ranged from 1.88 × 10^5^ to 4.04 × 10^5^.

### 2.3. Mesenchymal Stromal Progenitor Cell-Associated Markers

**Figure 6:**
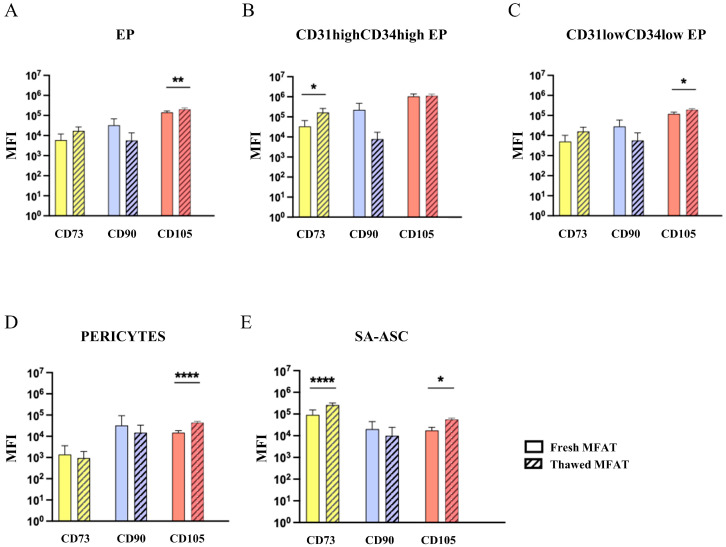
Expression of the mesenchymal stromal progenitor cell-associated markers. CD73, CD90, and CD105 cell surface markers on endothelial progenitors (**A**); the CD31highCD34high (**B**) and CD31lowCD34low (**C**) subsets of endothelial progenitors (EP), pericytes (**D**), and supra-adventitial adipose stromal cells (**E**) in fresh MFAT (clear boxes) and frozen MFAT (dashed boxes) from the same six patients. The geometric mean fluorescence intensity (MFI) data are expressed as bars with standard deviation of six patients, and statistical analysis was performed using t-tests based on the normal distribution; *p*-values: (*) *p* < 0.05, (**) *p* < 0.01, (****) *p* < 0.0001.

The CD73, CD90, and CD105 cell surface markers associated with progenitor cell populations were analyzed (Figure 6). The expression of the CD73 marker was significantly higher on the CD31^high^CD34^high^ subset of EP and SA-ASC populations from thawed MFAT samples. The expression of the CD105 marker was significantly higher on EP, the CD31^low^CD34^low^ subset of EP, which represents a majority of the EP, pericyte, and SA-ASC cell populations from thawed MFAT samples.

### 2.4. Morphological Evaluation of Cryopreserved Adipose Tissue

**Figure 7:**
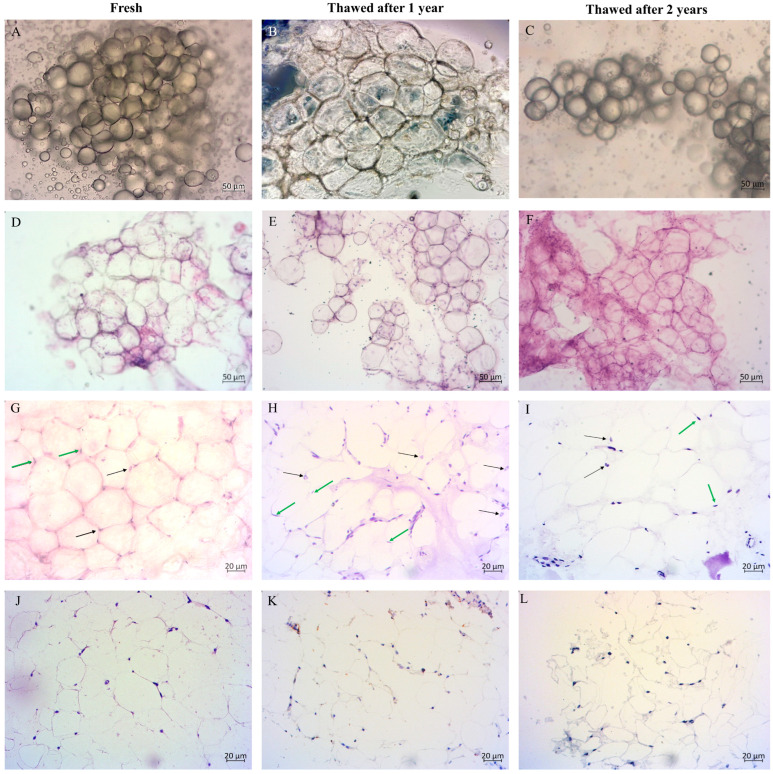
Morphology of subcutaneous adipose tissue (fresh) and MFAT (thawed). Micrograph of adipocyte suspension from fresh subcutaneous fat (**A**), and cryopreserved MFAT suspension after one year ((**B**), 10× objective) and after two years (**C**). The uniform size and spherical shape of the fat cells are clearly visible. White adipose tissue cells are among the largest cells in the body, reaching up to 100 μm in diameter. Smear micrograph of fresh adipose tissue and MFAT cells, both fixed with 10% formalin (H&E), also shows the uniform size and spherical shape of adipocytes. Other cells that are normally present in adipose tissue are also visible, such as fibroblasts, macrophages, neutrophils, and lymphocytes (**D**–**F**). Adipocytes from paraffin-embedded subcutaneous adipose tissue, as well as MFAT in paraffin-embedded gel matrix show the same morphology (H&E). Adipocytes appear clear in routine H&E-stained sections because cytoplasmic fat dissolves during tissue processing. The lipid droplet occupying the cytoplasm presses the adipocyte nucleus against the cell membrane, giving it a thin crescent shape (marked with green arrows). We observed several nuclei with a central vacuole (Lochkern) (marked with black arrows). Furthermore, very thin cell membranes and fibrous septa between the cells were visible (**G**–**I**). Immunohistochemical staining revealed that adipocytes in both samples did not show proliferation and were negative for Ki-67 (**J**–**L**); (n = 4).

Fresh and thawed AT show very similar morphological characteristics (Figure 7). Histologically equally processed subcutaneous AT (marked as fresh) served as a control for MFAT cryopreserved for one or two years (Figure 7A–C). Mature adipocytes contain a large, irregular central lipid droplet that occupies more than 90% of the cell volume, as is clearly visible in both fresh and cryopreserved AT stained with H&E (Figure 7D–I). Consequently, the cytoplasm, along with organelles such as mitochondria and the endoplasmic reticulum, as well as the nucleus, is compressed against the cell membrane, causing the nucleus to adopt a crescent shape (green arrows in Figure 7G–I). Morphologically, the cells from MFAT (thawed) exhibit characteristics of healthy mature adipocytes (fresh), including uniformity in the size and appearance of nuclei, preserved membranes, and maintained fibrous septa (Figure 7G–I). Additionally, some cells showed nuclei with a central vacuole (Lockhern cells) (black arrows in Figure 7G–I). Because of the crescent shape, the nucleus of normal adipocytes in histological sections sometimes appears to have a vacuole. Immunohistochemical analysis of Ki-67 was below 1% (Figure 7J–L), with some positivity in fibroblasts and macrophages (not shown in the image).

### 2.5. Analysis of DMSO Residue in MFAT Preparation After Cryopreservation

In order to quantify the levels of residual DMSO in washes of MFAT preparation with 10% and 1% human plasma albumin (HPA), a reverse-phase high-performance liquid chromatography (RP HPLC) method was developed. A calibration curve based on the peak-area ratio against DMSO standard concentrations was used for the calculation of the residual DMSO concentration in the washing samples of MFAT.

After the addition of 10 mL of 10% HPA to thawed MFAT samples, and before the aqueous part was separated from the AT, the nominal concentration of DMSO was 0.76%. The average concentration of DMSO in the first wash, from analysis of seven independent samples, was 0.762 ± 0.121% (average ± 95% confidence interval), which equals the nominal concentration and indicates the removal of 90% DMSO already in the first wash. DMSO concentration in the second wash with 1% HPA was 0.024 ± 0.009%, which indicates additional removal of 2–3% of DMSO (Table 1).

### 2.6. Microbiological Evaluation of MFAT Preparation

Microbiological analysis of all samples (biological and environmental) during storage, thawing, and preparation for clinical application confirmed that they were 100% sterile at all times.

## 3. Discussion

**Figure 8:**
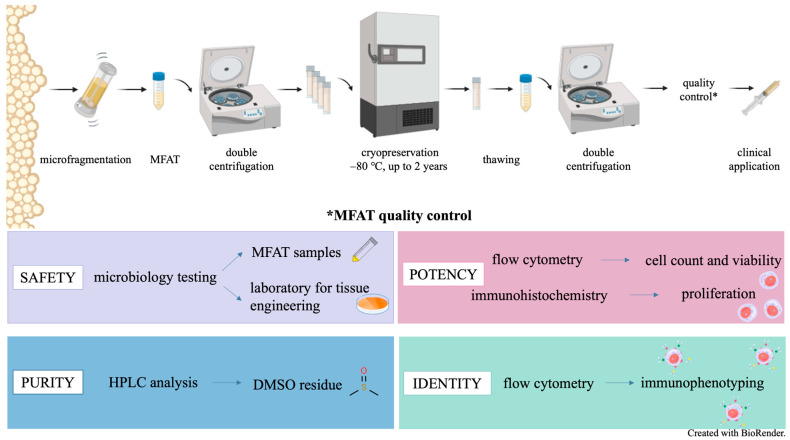
Methods used in evaluation of cryopreserved microfragmented adipose tissue (MFAT) for use in autologous clinical applications. * Overview of quality control methods. Created with BioRender.com.

**Figure 9:**
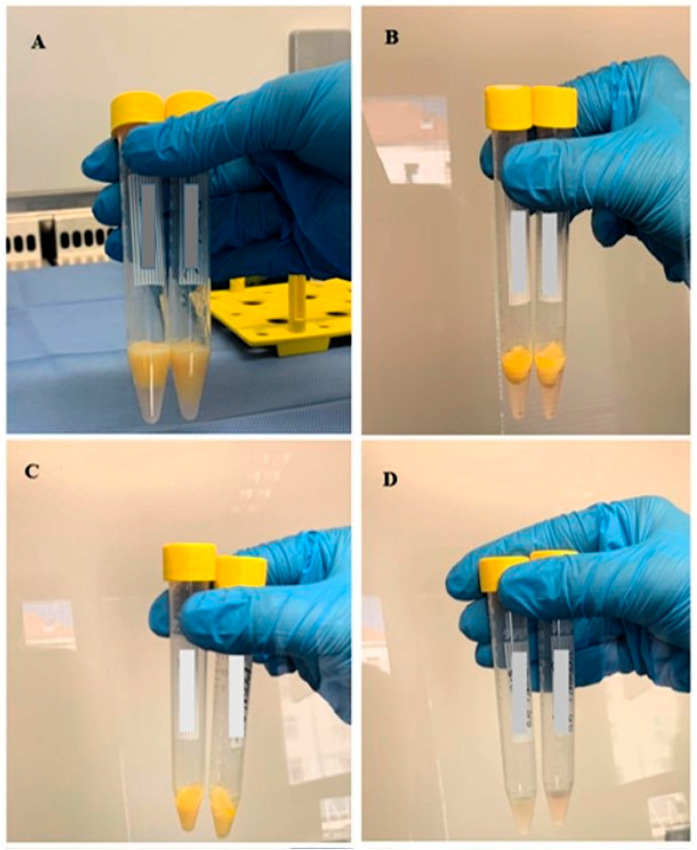
Cryopreservation of MFAT. Cryopreservation of microfragmented adipose tissue (MFAT) is a procedure that involves transferring adipose tissue into a conical tube (**A**), followed by centrifugation (200× *g*, 5 min at 22 °C) to separate the adipose from the aqueous part (**B**) in order to obtain the highest possible concentration of adipose tissue (**C**). The procedure is repeated three times. The aqueous part is sent for microbiological analysis (**D**).

**Figure 10:**
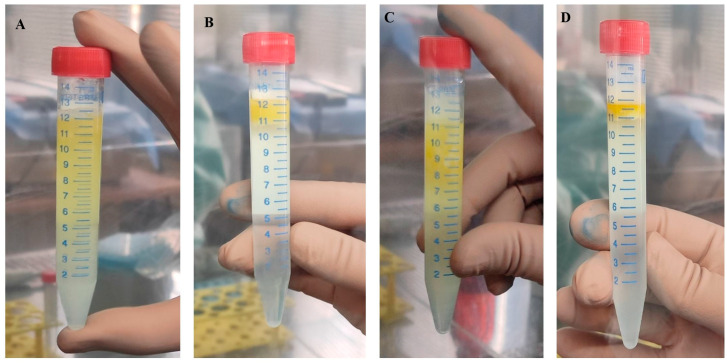
The procedure for the first and second washing of MFAT (microfragmented adipose tissue). The procedure involved the following steps: First Washing (**A**,**B**): Cryopreserved MFAT was first washed using 10% HPA in sterile physiological saline (0.9% NaCl). The process included gently mixing the tissue in the solution to ensure thorough removal of DMSO. After washing, the solution was carefully separated and sent for microbiological analysis and/or residual DMSO analysis. The MFAT was then centrifuged to concentrate the tissue and prepare it for the next washing step. Second Washing (**C**,**D**): After the first washing, MFAT underwent a second washing using 1% HPA in sterile physiological saline. This second washing aimed to further purify the tissue by removing any remaining DMSO residues and achieving a higher degree of sterility. The procedure involved repeated gentle mixing to ensure an effective removal of all remaining impurities. After the second washing, the solution was carefully separated and sent for microbiological analysis and residual DMSO analysis, and the MFAT was prepared for subsequent processing steps, such as clinical application. These steps are designed to ensure maximum purity and sterility of the MFAT, which is crucial for its safety and effectiveness in clinical practice [37].

**Figure 11:**
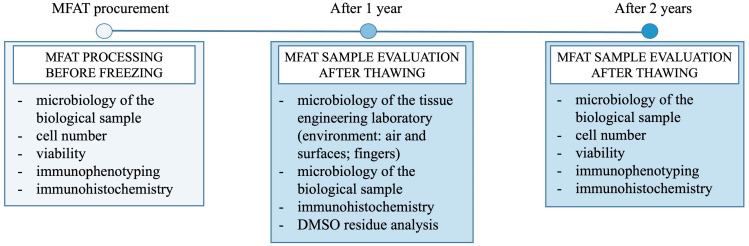
Overview of the timeline and methods used in implementing MFAT into routine clinical practice.

The impressive potential of the stem/progenitor cells of AT drives many researchers to explore its extensive applications in regenerative medicine. Since optimal results in cartilage regeneration may be achieved with multiple injections into the knee joint, it is beneficial to store small aliquots of AT. To make the process of OA therapy using MFAT as convenient as possible, cryopreservation is an essential step [19,20]. Our study (Figure 8) provides a transparent description of the preparation, cryopreservation, thawing, and washing processes of MFAT, supported by detailed images (see Figure 9 and Figure 10), which, to our knowledge, have not been previously presented in the literature. This manuscript emphasizes a rational and cautious approach during the validation process and introduces a safe method for cryopreservation of this tissue banking product. Nevertheless, for selected patients, we thawed one cryovial of MFAT from a batch of stored samples, after one or two years of storage at −80 °C, to perform quality control tests (Figure 11). This procedure represents a step towards integrating this therapy into the field of personalized and precision medicine.

Osteoarthritis primarily affects adults over the age of 50. Approximately 10% of people over the age of 55 suffer from painful, disabling knee osteoarthritis [21]. However, it can also occur in younger individuals, particularly if they have predisposing factors such as previous joint injuries, obesity, or genetic predisposition. Our samples were collected from 15 patients (aged 43–74 years, average age 60 years). Based on the values obtained for each method, it appears that the results are not strongly correlated with patient age. However, due to the small sample size, we need to interpret the results cautiously and avoid drawing conclusions related to age.

Our results related to the viability of live cells in fresh and thawed samples show that the viability was not significantly affected by the process of cryopreservation. These results are in accordance with the data published by Favaretto et al. [20], who explored the viability of SVF in fresh and frozen AT. However, a significant difference was observed in the total cell count between fresh and thawed MFAT samples, demonstrating that approximately half of the cells were lost during the cryopreservation and/or thawing processes. The true reason for the 50% cell loss is unknown. It could be due to cell death, whether necrotic or apoptotic, or to cell loss during several centrifugation steps while thawing the MFAT samples. Cell type immunophenotyping evidenced a substantial decrease in the absolute and relative numbers of all the identified cell types. However, due to high intra- and inter-patient variations, a statistical significance was reached only for the CD31^high^CD34^high^ subset of EP, as well as for the increase of the “other cells” group (Figure 2 and Figure 3). Furthermore, an approximate 60% reduction in leukocytes was observed (but not statistically significant), resulting from repeated centrifugation and washing steps. These findings support the use of frozen adipose tissue, as the decreased proportion of blood cells reduces inflammatory responses and improves the efficacy of intra-articular application [2]. Therefore, although some cells were lost during the cryopreservation process, these results show that the viability and diverse cellular composition of the MFAT are preserved even after two years of storage in a tissue bank.

Mesenchymal stem cells should not be confused with mesenchymal stromal cells, despite sharing the same acronym. The stromal MSCs have been defined as plastic-adherent cultured cells capable of differentiating into mesodermal lineages and expressing mesenchymal markers such as CD73, CD90, and CD105, while lacking expression of CD45, CD34, CD14 or CD11b, CD79α or CD19, and HLA-DR surface molecules [22]. The stemness of MSCs must be rigorously proven using clonogenic assays to distinguish rare stem from heterogeneous stromal MSCs, and robust functional assays are required to verify the therapeutic mode of action evidencing stem or stromal MSC functionality [23]. Moreover, MSCs concurrently positive for CD73, CD90, and CD105 markers are extremely scarce and often below the detection limit of flow cytometry in native tissues, including adipose tissue. Similar to others, we were unable to detect CD73^+^CD90^+^CD105^+^ cells in either fresh or fresh-frozen MFAT samples after thawing. This is consistent with other studies demonstrating the elusive nature of CD73^+^CD90^+^CD105^+^ cells in freshly isolated stromal cells from AT compared to cultured MSCs [24], particularly due to the weak expression of CD105 in the former [18,25]. Importantly, pericytes, and SA-ASC are considered the in vivo precursors of MSCs [26,27,28], located in perivascular and supra-adventitial regions around small blood vessels, respectively. Together with EP located in the luminal layer, they represent the main stem/progenitor cell populations in adipose tissue, exhibiting a highly mesenchymal phenotype [29]. Therefore, we investigated the expression of mesenchymal surface markers CD73, CD90, and CD105 on progenitor cell populations and subpopulations before and after storage. We found significant differences in the expression profile of MSC-associated markers between fresh and thawed MFAT samples, including an increase in CD73 expression on the CD31^high^CD34^high^ subset of EP and SA-ASC, as well as an increase in CD105 expression on EP, the CD31^low^CD34^low^ subset of EP, pericytes and SA-ASC after MFAT cryopreservation. It remains unclear whether these changes reflect documented cell loss or the upregulation/downregulation of these markers during the cryopreservation process. Nonetheless, additional experimental studies are required to determine the biological relevance of the observed changes.

Comparison of the histological appearance of fresh and thawed AT reveals the expected adipocyte morphology with no differences between samples. Since complete morphology of the cells is not visible on smears, clearer observation of their structure requires creating cell sections by embedding the cells in a cytoblock. In our research, examination of the morphology of MFAT did not reveal any pathological changes, such as noticeable variations in cell size and shape, thickened fibrous septa between cells, or disintegration of the cell membrane, which are clear indicators of adipocyte death (apoptosis or necrosis) [30]. Regarding proliferation, low expression of Ki-67 is typical for AT because mature adipocytes normally do not proliferate. Additionally, we did not observe any changes related to hyperplasia and hypertrophy [31].

We have noticed that the washing of DMSO has not been adequately addressed in previous studies, highlighting the importance of our findings regarding the safety and quality control of cryopreserved MFAT. In our research, the average DMSO concentration in the MFAT washing fluids, which is nearly equal to the nominal concentration, indicates that DMSO was successfully removed in the first wash. The second wash further removed any remaining traces of DMSO. Our results showed that effective removal occurred during the first washing. However, for safety and a gradual transition from a 10% HPA solution to a 1% HPA solution for MFAT application, a second wash is advisable. The maximum recommended dose of DMSO is 1 g/kg of the recipient’s body weight due to dose-dependent side effects, including nausea, vomiting, diarrhea, rashes, bronchospasm, headache, and cardiovascular changes [32]. Additionally, because DMSO is a cytotoxic and permeable cryoprotectant, its removal can be challenging [33]. Since we successfully removed approximately 90% of the added DMSO using a two-step washing procedure, the residual levels remained well below the maximum recommended dose, thereby ensuring the safety and quality of MFAT.

Microbiological sterility is crucial for clinical applications. Badowski et al. evaluated the effect of prolonged cryopreservation and observed some microbiological contamination, likely due to the procurement method [19]. Our results show 100% sterility in microbiological testing throughout all phases of MFAT processing. We obtained a microbiologically sterile MFAT product without performing a decontamination procedure. First, the use of the Lipogems^®^ [34] closed system allows for the safe collection of tissue in the operating room, and second, tissue processing continues in the tissue bank under aseptic conditions.

Based on the validation results, we have initiated an ongoing clinical study to evaluate the therapeutic potential of cryopreserved MFAT in patients with knee OA at baseline and six-month follow-up using validated clinical questionnaires such as KOOS (Knee Injury and Osteoarthritis Outcome Score), WOMAC (The Western Ontario and McMaster Universities Arthritis Index), and VAS (Visual Analog Scale for pain). Preliminary clinical follow-up of the first two patients treated with thawed autologous MFAT (unpublished data) demonstrated a notable reduction in joint effusion and stiffness—symptoms commonly attributed to synovial inflammation, as was particularly seen in increased KOOS Symptoms and decreased WOMAC Stiffness scores. These early findings suggest that the immunomodulatory and anti-inflammatory effects typically associated with MSCs (we published numerous articles describing the functional activities of those cells [35,36]) may be preserved following cryopreservation. However, larger clinical studies will be necessary to confirm these effects and establish their significance.

Furthermore, we would like to highlight a limitation that we consider important to mention. Due to the small number of samples, we believe further testing on a larger sample size is necessary, which we plan to pursue in future research.

## 4. Materials and Methods

### 4.1. Study Design

The research on cryopreserved MFAT is part of the project titled “Clinical and Molecular Phenotyping of Osteoarthritis: A Personalized Approach to Diagnosis and Treatment” in collaboration with the St. Catherine Specialty Hospital and the Sestre milosrdnice University Hospital Center in Zagreb, Croatia. With this project, a cooperation agreement was signed, and approval was obtained from the Ethics Committee of Sestre milosrdnice University Hospital Center to process, aliquot, and store MFAT in quarantine at −80 °C until test results are available. After receiving the results of sterile microbiological tests for aerobic and anaerobic microorganisms, as well as molds, MFAT samples were stored in a non-quarantine freezer until clinical application (Figure 8). Additionally, immunophenotyping, cell count, and viability assessments were performed before storage. After one year, some patients were to receive cryopreserved MFAT, but before clinical application, cryoprotectant DMSO had to be removed and additional analyses were performed: the cell count and viability assessments, as well as microbiological testing and immunohistochemical analysis. The success of the DMSO removal procedure was monitored. By participating in this project, our goal was to develop a new tissue banking product with a specific application in clinical practice based on the principles of personalized and precision medicine.

### 4.2. Initial Examination and Procurement

If the surgeon determined advanced degenerative changes in the knee cartilage (grades 2–3) and considered it necessary to proceed with treatment using MFAT, it was necessary to contact the responsible person at the Tissue and Cell Bank (TCB) to arrange details related to the storage of MFAT and to obtain the appropriate documentation. After the initial examination of the patient with knee osteoarthritis and the procurement of lipoaspirate using Lipogems^®^ (Lipogems International S.p.A, Milan, Italy) at the St. Catherine Specialty Hospital, the transportation and receipt of MFAT were carried out to the TCB.

### 4.3. Cryopreservation

The MFAT storage procedure started with transferring the sample from syringe to a labelled 15 mL conical tube, which was carried out in a class II microbiological safety cabinet (KTB-NS III, Klimaoprema, Samobor, Croatia). The syringe with and without MFAT was weighed on a precision balance (Radwag, PS 1000.R2 Precision balance, Radom, Poland), and the weight was recorded. After that, the sample was centrifuged three times at 200× *g* for 5 min at 22 °C (Eppendorf SE, Centrifuge 5810R, Hamburg, Germany) (Figure 9). Following each centrifugation, it was necessary to separate the aqueous part extracted from the MFAT into separate 15 mL tubes. These tubes were then sent for microbiological analysis to check for sterility at the accredited medical laboratory in the Croatian Institute of Transfusion Medicine, Zagreb, Croatia. An equal volume of freezing medium (1:1) was added to the obtained volume of MFAT, consisting of 10% DMSO (AL.CHI.MI.A. S.R.L., Ponte San Nicolo, Italy), 40% human plasma albumin (HPA) (200 g/L Kedrion, Barga, Italy), and 50% Dulbecco’s phosphate-buffered saline (DPBS) (Sigma-Aldrich, Saint Louis, MO, USA). The mixture of MFAT and freezing medium had to be thoroughly resuspended and distributed into 2 mL cryotubes. Depending on the volume of the received MFAT, an appropriate number of cryotubes was prepared. Each cryotube was filled with approximately 1.8 mL of the MFAT and freezing medium mixture (0.9 mL of MFAT and 0.9 mL of freezing medium). Before storage, the cryotubes were labeled with the name, surname, date of birth, date of storage, and the initials of the personnel who performed the storage procedure. Additional information about the stored MFAT can be found in the accompanying documentation. The expiration date was recorded in the accompanying documentation of the cryopreserved MFAT, which was created after the results of all analyses were available. The cryotubes, properly sealed, were stored in a cooled container, and the freezing procedure had to be carried out without delay. The MFAT in the freezing medium was placed in a Mr. Frosty (Thermo Scientific™, Waltham, MA, USA) with isopropanol (which is kept at room temperature) and then immediately stored in a cryobank at −80 °C in a quarantine freezer until microbiological results are received. After acceptable results are obtained, the MFAT is stored long-term in a non-quarantine freezer until clinical application.

### 4.4. Thawing and Preparation of MFAT Batch

In one procedure of liposuction, usually 10 mL of MFAT was obtained, which resulted in a batch of five cryovials. Approximately one month before clinical application, one cryovial from the batch was thawed, washed, and sampled for evaluation of microbiological sterility, cell number, and viability, as well as of the success of DMSO removal. The thawing and washing procedure was set as follows. Before starting the thawing process, 1% and 10% solutions of human plasma albumin (1% HPA and 10% HPA) must be prepared. The cryovial with MFAT was then immersed in a glass beaker with slightly warmed sterile water, and it thawed within one minute. The whole content was transferred into a sterile 15 mL tube, and 10 mL of 10% HPA solution was added for the first wash of thawed MFAT (Figure 10). The content was gently resuspended and homogenized. Then, it was centrifuged at 300× *g* for 4 min at 4 °C (Heraeus Labofuge 400 R, Hanau, Germany), separating the aqueous part from the MFAT, which became clearly visible on the surface. Using a sterile needle and a 5 mL syringe, the aqueous part below the MFAT was completely removed and afterwards sent for microbiological sterility and DMSO content determination (only MFAT remained in the tube).

After the first wash, a second wash of MFAT was performed in the same tube by adding 10 mL of 1% HPA solution without resuspending. The tube was manually turned several times to homogenize the contents, as was done in the first wash. After the second wash, the tube with MFAT was centrifuged again under the same conditions. Then, it was necessary to repeat the procedure of removing the aqueous part below the adipose tissue with a needle and syringe and to send it for microbiological sterility and DMSO content determination. Washed MFAT from cryovials of the same batch was transferred into a single 50 mL tube.

The target number of cells to be administered in a single intra-articular injection was approximately 1–2 × 10^6^ cells. If 5 cryovials were stored, one was used for quality control tests, and the remaining 4 cryovials, after double washing, contained “concentrated” MFAT, which typically amounted to about 3.5 mL. To this volume, an equal amount of 1% HPA solution (in a 1:1 ratio) was added to ensure that the total volume in the syringe, which would be injected into the patient’s knee, did not exceed 7 mL. After aspiration, the needle was replaced with a new one, and the syringe was placed in labelled sterile zip bags. The label contained the following information: patient’s name and surname, corresponding ID/code, number of viable cells, total volume of MFAT solution, and date of preparation.

### 4.5. Quality Control

A critical aspect of quality control pertains to the safety of the preparation (Figure 11), which is primarily ensured through microbiological sterility by mandatory testing of each batch (one aliquot is taken from the batch). Microbiological analyses of the tissue rinsing solutions must be performed before freezing and after thawing of one cryotube. In the case of positive microbiological results, the tissue must be discarded. Additional testing is performed immediately before clinical application (Table 2). For testing the morphological characteristics of adipose tissue, native smears on glass slides and a suspension of adipocytes in 10% buffered formalin were used. The goal is to obtain information on the preservation of the MFAT structure and on the individual adipocytes that have undergone processing, storage with a cryoprotectant, and thawing from MFAT samples of 15 patients (age 43–74 years, average 60 years). Cell viability and cell count before and after storage were analyzed by flow cytometry as part of the recovery or potency testing. In addition to the mentioned flow cytometry analyses, immunophenotyping of MFAT before and after storage was conducted as part of the identity. In addition, as part of the quality control, the success of DMSO removal was monitored by DMSO quantification in both washes using an in-house RP HPLC method. After the validation process, the shelf life of MFAT was determined based on all the mentioned quality control results, with special emphasis on analyses performed after long-term storage (two years). Based on the described procedures and validation results, we recommend that standard quality control analyses, including cell count, viability, immunophenotyping, and microbiological testing, be routinely performed after long-term storage and prior to clinical application. Additional analyses, such as DMSO residue quantification and histological assessment of MFAT, should be performed only during the validation process.

#### 4.5.1. Microbiological Testing

The traceability of the process is recorded during all stages of reception, processing, storage, thawing, transport, and delivery of MFAT. During all these procedures, microbiological control of the sterility of the working environment within the laboratory is performed, including the fingers of the personnel conducting these procedures as well as the biological samples. Sterility was tested in an accredited medical laboratory according to ISO standard 15 189 [38] at the Croatian Institute of Transfusion Medicine in Zagreb, Croatia. Biological samples (rinse solutions, MFAT samples) were analyzed using the direct inoculation method with a 14-day incubation period. Thioglycollate broth with resazurin (Biomerieux, Craponne, France) was used for the cultivation of anaerobic bacteria at 37 °C, while soybean-casein digest medium (Biomerieux, Craponne, France) was used for the cultivation of fungi and aerobic bacteria at 22 °C.

#### 4.5.2. Immunohistochemistry

The MFAT cell suspension was mixed with double the volume of 10% formalin and left to fix the cells for at least six hours. After fixation, the cells were spread on positively charged Superfrost Plus adhesive microscope slides (VWR European Cat. No. 631-0108, Leuven, Belgium), air-dried for 12 h, and stained with hematoxylin and eosin (H&E). The remaining formalin-fixed MFAT cells were processed into cell blocks and embedded in paraffin for further analysis. Agar gel was used as a matrix to support the cells during tissue processing.

To prepare 8% agar gel, 0.8 g of agar powder (Agar-Agar, BioScience Grade, CAS No.: 9002-18-0, Columbus, OH, USA) was dissolved in 10 mL of deionized water through gradual heating in a microwave with constant stirring, ensuring it did not boil. Once dissolved, 1–2 mL of the agar was poured into a glass tube and allowed to cool to 70 °C before adding an equal amount of the cell suspension. After gently mixing the cells to achieve a uniform mixture of agar and cells, the gel was cooled in the refrigerator for several hours, preferably overnight.

The next day, the agar was carefully detached from the glass tube and transferred to histological cassettes (Sakura, Tissue-Tek Uni-cassette system; Heerlen, The Netherlands) for paraffin processing in a tissue processor. To prevent cell leakage through the cassette holes during processing, the gel with cells was placed between two foam pads (Biopsy Foam Pads; Simport Scientific; Beloeil, Canada) in the histology cassettes. After routine processing, the cells were embedded in paraffin blocks.

For further analysis, a few cross-sections were cut from the cell block using a Zeiss microtome (Hyrax S50, Kuala Lumpur, Malaysia). One section was stained with H&E to examine cell morphology, while another was placed on positively charged immuno-slides for immunohistochemical staining. Antigen retrieval was performed in a high pH Tris buffer (9.0) at 97 °C for 20 min using a PTLink device (Dako, Hovedstaden, Denmark). The staining was carried out using an Autostainer Link48 automated instrument (Dako, Denmark) with Ki-67 primary antibody (MIB-1, Dako, Denmark, dilution 1:100) to monitor cell proliferation.

After antigen unmasking, the Ki-67 primary antibody was applied to the slides for 30 min. The slides were then washed with buffer, and the secondary antibody from the DAKO EnVisionTM FLEX/HRP detection kit (DAKO K8000) was applied for 30 min at room temperature. Finally, hematoxylin was used for counterstaining, and representative micrographs were captured using a Carl Zeiss camera with ZEN Lite 2.3 software on a Zeiss Axio Star microscope. Adipose tissue, taken less than two hours after surgery (a short period of cold ischemia), processed in the same way and embedded in a tissue paraffin block, as well as a cytoblock, served as a control. This tissue is referred to in the text as fresh adipose cells.

#### 4.5.3. Reverse Phase High-Performance Liquid Chromatography (RP HPLC) for Estimation of Residual DMSO

The RP HPLC analysis was performed using a Shimadzu Prominence LC-20AD system with a UV detector SPD-M20A, an autosampler SIL-20AC HT, and LabSolutions 5.99 software (Kyoto, Japan). Chromatographic separation was carried out on a HYPERSIL ODS-2 C18 column (5 μm, 125 × 4 mm) equipped with a guard cartridge packed with the same stationary phase (Thermo Fisher Scientific, Waltham, MA, USA). The mobile phase was composed of water (A) and methanol (B) in a ratio of 95:5 (*v*/*v*). RP HPLC analysis was conducted in isocratic mode at a flow rate of 1 mL/min and room temperature. Detection was performed at a wavelength of 207 nm. The injection volume was 20 µL, and the total run time was 5 min.

A concentrated stock solution (10% DMSO) was prepared by adding 50 μL of DMSO to 450 μL of the mobile phase. The working standard solution (0.1% DMSO) was prepared by diluting the stock solution 10-fold with the mobile phase and was used to prepare ten standard samples covering a concentration range of 0.0000975% to 0.05%. Samples from the first wash of MFAT were diluted 100-fold with the mobile phase, while samples from the second wash of MFAT were diluted 50-fold. The DMSO standards and the samples were centrifuged (Eppendorf, Hamburg, Germany) at 3000× *g* for 20 min and then transferred to vials for RP HPLC analysis. Both standards and samples were analyzed in duplicate. The DMSO quantification was based on a DMSO calibration curve.

#### 4.5.4. SVF Cell Isolation for the Flow Cytometry Analyses

Thawed and washed MFAT samples were mixed 1:1 with 0.2% collagenase type I in Dulbecco’s Modified Eagle Medium (D-MEM) (both from Sigma-Aldrich, Saint Louis, MO, USA) and digested in a shaking bath at 37 °C for 30 min. After 1:2 dilution with 10% heat-inactivated fetal bovine serum (Biosera, Nuaille, France) in D-MEM, samples were filtered through a 100 μm cell strainer (BD Falcon, Corning, New York, NY, USA) and centrifuged at 500× *g* for 10 min at room temperature (RT). Supernatants were discarded, and the cell pellet was resuspended in 1 mL of the VersaLyse solution (Beckman Coulter, Miami, FL, USA). After 10 min, samples were filtered through a 40 μm cell strainer (BD Falcon, Corning, NY, USA), centrifuged at 500× *g* for 5 min at RT, and the cell pellet resuspended in 500 μL phosphate-buffered saline (PBS; Sigma-Aldrich, Saint Louis, MO, USA).

#### 4.5.5. Flow Cytometry Immunophenotyping of SVF from MFAT

The SVF cells isolated from both fresh and thawed MFAT (recommended concentration: 100 μL cell suspension per tube with 3 × 10^6^ cells/mL) were first stained using Live/Dead Yellow Fixable Stain (ThermoFisher, Waltham, MA, USA) for 15 min at RT protected from light, washed with PBS, and centrifuged at 500× *g* at RT. The supernatant was discarded, and the cell pellet was resuspended in 100 μL and transferred to a Duraclone SC dry reagent prototype tube (Beckman Coulter, Miami, FL, USA) that enables the identification of the MSC subpopulations based on the use of antibodies specific for the cell surface markers (CD90, CD73, CD34, CD146, CD105, CD45, CD31, CD14, CD19). The samples were gently mixed and incubated for 15 min at RT, protected from light. Upon centrifugation at 500× *g* at RT in PBS, the supernatant was discarded, and the cell pellet was resuspended in 500 μL of 0.1% paraformaldehyde (Electron Microscopy Sciences, Hatfield, PA, USA) in PBS for the sample fixation. Acquisition of the samples was performed on the DxFlex flow cytometer (Beckman Coulter, Miami, FL, USA). The Flow Cytometry Standard (FCS) data files were analyzed using the FlowLogic 8.6 software (Inivai Technologies, Mentone, Australia).

#### 4.5.6. Flow Cytometry Determination of SVF Cell Viability

For the cell viability determination, 100 μL of the SVF cell suspension was stained with 2 μL of ViaStain AOPI Staining Solution (Nexcelom Bioscience LLC., Lawrence, MA, USA). After 5 min, 500 μL of PBS and 20 μL of the 1000×-diluted DRAQ5 dye (BioStatus, Loughborough, UK) were added, and the samples were incubated for 5 min at RT, protected from light. Acquisition of the samples was performed on the DxFlex flow cytometer (Beckman Coulter, Loveland, CO, USA) immediately upon staining, and the FCS data files were analyzed using the FlowLogic software V.8 (Inivai Technologies, Victoria, Australia).

#### 4.5.7. Flow Cytometry Determination of SVF Cell Count

For the cell count determination, 100 μL of cell suspension was diluted with 1 mL of PBS (Sigma-Aldrich). Then 20 μL of the 1000x-diluted DRAQ5 dye (BioStatus) was added to each sample. Samples were gently vortexed and proceeded to acquisition on the DxFlex flow cytometer (Beckman Coulter). Before acquisition, 100 μL of FlowCount fluorospheres (Beckman Coulter) was added, and the samples were firmly vortexed. The FCS data files were analyzed using the FlowLogic software (Inivai Technologies). The cell count was calculated according to the count bead manufacturer’s instructions from the following formula:

Absolute Count (cells/μL) = Total Number of Cells Counted/Total Number of Fluorospheres Counted × Flow-Count Fluorospheres Assayed Concentration.

The final cell count was calculated and presented as cell number per tissue gram.

### 4.6. Statistics

Statistical analysis was performed using parametric tests stated under the figure(s) based on the normality test for normal distribution (GraphPad Prism 10 for macOS; GraphPad Software 10.3.1, Inc., San Diego, CA, USA). A *p*-value < 0.05 was considered statistically significant.

## 5. Conclusions

This paper presents the quality control procedure conducted during the validation of cryopreserved MFAT, ensuring a high-quality preparation ready for routine clinical practice in patients with OA. Histological analysis of cryopreserved MFAT reveals well-preserved mature adipocytes, characterized by intact cell membranes and consistent size and appearance of nuclei. The microbiological sterility of MFAT before and after freezing indicates a high level of safety for this preparation. Supporting this is the fact that double washing of MFAT achieved a minimal amount of residual DMSO, ensuring the high purity of the preparation. In fresh MFAT, immunophenotyping characterized the composition of the SVF with a significant proportion of endothelial progenitors and pericytes. Comparing cell count and viability before and after cryopreservation revealed no statistically significant difference in cell viability, although a lower number of cells was observed after thawing, most likely due to multiple centrifugation and washing steps during the freezing and thawing process. However, in the intra-articular application of cryopreserved MFAT, this method allows for the calculation of the approximate number of cells in each aliquot/syringe, enabling the target number of cells to be administered into the knee. Our results show that cryopreserved MFAT retains its structural integrity with slight changes in cellular composition but with preserved cellular viability even after two years of storage at −80 °C, suggesting it is a reliable autologous therapeutic option for intra-articular application in patients with osteoarthritis.

## Figures and Tables

**Table 1:** Obtained amounts of % DMSO (*v*/*v*) in the washes of MFAT. The calculated values from the calibration curve represent the mean value of the amount of DMSO in the samples (which were applied in duplicate). The nominal concentration of DMSO in samples before the aqueous part was separated from the adipose tissue was 0.76% DMSO (n = 7).

Samples	Patient ID	Samples Procedure	Specimen	% DMSO
Sample 1	023	1. washing of MFAT	10% HPA	0.7191
		2. washing of MFAT	1% HPA	0.0226
Sample 2	028	1. washing of MFAT	10% HPA	0.7944
		2. washing of MFAT	1% HPA	0.0271
Sample 3	033	1. washing of MFAT	10% HPA	0.5594
		2. washing of MFAT	1% HPA	0.0156
Sample 4	040	1. washing of MFAT	10% HPA	0.6274
		2. washing of MFAT	1% HPA	0.0129
Sample 5	036	1. washing of MFAT	10% HPA	0.8671
		2. washing of MFAT	1% HPA	0.0354
Sample 6	047	1. washing of MFAT	10% HPA	0.8802
		2. washing of MFAT	1% HPA	0.0382
Sample 7	051	1. washing of MFAT	10% HPA	0.8881
		2. washing of MFAT	1% HPA	0.0410

**Table 2:** Analysis of representative samples during validation and clinical application of MFAT.

Analysis	Sample Type	Specimen
Microbiological sterility	Laboratory environment	Air, contact, fingers
	MFAT	Adipose tissue
	Aqueous phase of MFAT	1. washing of MFAT 10% HPA
		2. washing of MFAT 1% HPA
DMSO residue	Aqueous phase of MFAT	1. washing of MFAT 10% HPA
		2. washing of MFAT 1% HPA
Immunohistochemistry	MFAT	Adipose tissue
Cell viability	MFAT	Adipose tissue
Cell number	MFAT	Adipose tissue
Immunophenotyping	MFAT	Adipose tissue

## Data Availability

The data sets generated during this study are available from the corresponding author on request.
